# Effectiveness of Pharmacological Treatments for Adult ADHD on Psychiatric Comorbidity: A Systematic Review

**DOI:** 10.3390/jcm14248848

**Published:** 2025-12-14

**Authors:** Beniamino Tripodi, Manuel Glauco Carbone, Irene Matarese, Roberta Rizzato, Filippo Della Rocca, Francesco De Dominicis, Camilla Callegari

**Affiliations:** 1 Department of Medicine and Surgery, Division of Psychiatry, University of Insubria, Viale Luigi Borri 57, 21100 Varese, VA, Italy; beniamino.tripodi90@gmail.com (B.T.); roberta-rizzato@libero.it (R.R.);; 2 Department of Clinical and Experimental Medicine, Division of Psychiatry, University of Pisa, Via Roma 57, 56100 Pisa, PI, Italy; 3 Department of Mental Health and Addictions, Division of Psychiatry, ASST Crema, Via Largo Ugo Dossena 2, 26013 Crema, CR, Italy; 4 VP Dole Research Group, G. De Lisio Institute of Behavioural Sciences, Via di Pratale, 3, 56121 Pisa, PI, Italy; 5 Departmental Faculty of Medicine, Saint Camillus International University of Health and Medical Sciences, Via di Sant’Alessandro 8, 00131 Rome, RM, Italy; 6 Addiction Research Methods Institute, World Federation for the Treatment of Opioid Dependence, 225 Varick Street, Suite 402, New York, NY 10014, USA; 7 Department of Surgical, Medical, Molecular and Critical Area Pathology, Clinical and Health Psychology, University of Pisa, Via Roma 57, 56100 Pisa, PI, Italy; irenematarese@gmail.com; 8 Addiction Unit, Department of Mental Health and Addictions, ASL5 Liguria NHS, Via Dalmazia 1, 19124 La Spezia, SP, Italy; 9 Department of Mental Health, Division of Psychiatry, U.S.L. Umbria 2, Via San Carlo 3, 06049 Spoleto, PG, Italy; francesco.dedominicis@gmail.com

**Keywords:** attention deficit and hyperactivity disorder, ADHD, bupropion, atomoxetine, methylphenidate, lisdexamfetamine, affective disorders, anxiety disorders, personality disorders, substance use disorders, disruptive disorders

## Abstract

**Background:** Attention-Deficit/Hyperactivity Disorder (ADHD) in adults is frequently accompanied by psychiatric comorbidities that worsen outcomes and complicate treatment. Pharmacological management is central in care, yet its impact on co-occurring disorders remains uncertain. This systematic review evaluated the effectiveness of commonly prescribed medications for adult ADHD (methylphenidate, atomoxetine, bupropion, and lisdexamfetamine) on comorbid mood, anxiety, personality, and substance use disorders. Tricyclic antidepressants were also included in the search strategy; however, no eligible adult studies assessing imipramine or desipramine in patients with ADHD and psychiatric comorbidity were identified. **Methods:** A systematic search of the literature was conducted to identify studies examining these medications in adults with ADHD and at least one psychiatric comorbidity. Eligible studies reported clinical outcomes for both ADHD symptoms and the co-occurring disorder. Data were extracted and narratively synthesized, with particular attention paid to treatment effects and sources of heterogeneity. **Results:** Across the included studies, pharmacological treatments consistently improved core ADHD symptomatology. Their effects on psychiatric comorbidity were more variable. Some evidence suggested beneficial outcomes for selected anxiety disorder subtypes and for features of Cluster B personality disorders, possibly related to reductions in emotional dysregulation and impulsivity. Findings regarding substance use disorders were mixed: several studies reported reduced craving or substance use, but long-term stabilization was inconsistent. Marked heterogeneity in study design, populations, and outcome measures limited comparability. **Conclusions:** Current pharmacological treatments for adult ADHD show reliable efficacy for core symptoms but inconsistent benefits across comorbid psychiatric conditions. While targeted improvements may occur in specific domains, the evidence base is insufficient to define optimal long-term strategies for adults with ADHD and complex comorbidity. Rigorous, longitudinal studies are needed to clarify medication effects on distinct comorbid profiles and to inform integrated treatment planning.

## 1. Introduction

Attention-Deficit/Hyperactivity Disorder (ADHD), as defined by the Diagnostic and Statistical Manual of Mental Disorders—5th edition, is characterized by persistent patterns of inattention and/or hyperactivity–impulsivity that deviate from developmentally appropriate levels [1]. These symptoms typically emerge in childhood, often manifesting as age-inappropriate levels of inattention, impulsivity, and hyperactivity in children and adolescents.

This clinical presentation is often correlated with a delay in the development of executive functions, a set of cognitive skills that allow us to plan, focus, and manage our actions effectively. These functions encompass a range of cognitive abilities, including *attention* (sustaining focus on tasks and filtering distractions), *working memory* (holding and manipulating information in mind), and *inhibition* (controlling impulses and resisting distractions). Furthermore, executive functions play a crucial role in *organization* (planning and sequencing of information and tasks), *time management* (estimating and allocating time effectively), and *emotional regulation* (managing emotions and responses). Other key executive functions include *flexibility* (the capacity to adjust to changing demands and shift perspectives) and *goal-directed behavior* (setting goals, planning steps towards their achievement, and maintaining motivation over time) [2,3].

Challenges with executive function are often more overt in childhood and adolescence, making it difficult for individuals to meet academic and social demands. However, as individuals with ADHD mature, they often develop coping mechanisms and strategies to compensate for these challenges. These strategies might include increased effort, specific organizational tools, or reliance on external structures for support. While these adaptations can be beneficial, they can also mask underlying executive function difficulties, making identification and diagnosis in adulthood more complex [4].

Adding to these challenges, emotional dysregulation (ED) is frequently observed in individuals with ADHD [5]. Characterized by extreme emotional instability and reactivity, ED is often present from childhood but can escalate during adolescence and adulthood as external demands increase (e.g., social, work, academic, and familial pressures). This heightened emotional reactivity can manifest as intense frustration, irritability, anger, sadness, or anxiety, making it challenging to manage daily life effectively [6]. While ED is not exclusive to ADHD and can be present in other neurodevelopmental disorders, such as autism spectrum disorder (ASD), or mood disorders like bipolar spectrum disorders, it is a significant symptom for many individuals with ADHD, impacting their relationships, academic or career pursuits, and overall well-being [7]. Notably, in females, ED may be the primary clinical manifestation of ADHD, potentially leading to underdiagnosis if not adequately recognized [8].

While a burst of energy and a struggle to sit still might be the most visible signs in younger individuals, these core symptoms often persist into adulthood, presenting ongoing challenges across the lifespan [9]. As children with ADHD grow up, their symptoms may fade, persisting in a sub-clinical form in 35% of cases and partially affecting their functioning. However, in 15% of cases, the symptomatic expression remains unmodified, leading to more significant ongoing challenges [10]. Even when symptoms lessen, adults with ADHD often experience these core challenges in different ways. For example, hyperactivity may not always translate to a flurry of movement but rather a persistent feeling of restlessness or an inability to truly unwind and engage in quiet activities. This internal sense of hyperactivity can also manifest as impulsivity, leading individuals to speak their minds, sometimes interrupting others, due to difficulty filtering thoughts and impulses. This impulsivity, a defining characteristic of ADHD, often results in acting without fully considering potential consequences. Delaying gratification can feel nearly impossible, and patience, especially in the face of frustration, can wear thin quickly [11,12,13].

The diagnosis of ADHD in adulthood presents a significant challenge due to the complex interplay of several factors. The heterogeneous nature of ADHD often leads to missed or inaccurate diagnoses during childhood and adolescence [14]. Further complicating the diagnostic process is the dynamic nature of ADHD, which manifests as evolving psychopathological phenotypes throughout an individual’s lifespan. Individuals who eventually receive an ADHD diagnosis in adulthood often exhibit a history marked by interpersonal difficulties, academic and occupational underachievement, and a persistent pattern of seeking novel and stimulating experiences that provide fleeting gratification [15]. Adding to the complexity is the high prevalence of comorbid neuropsychiatric disorders in this population. Studies indicate adults with ADHD are 4 to 9 times more likely to experience co-occurring mental health disorders than the general population [16]. Specifically, over 80% of adults with persistent ADHD experience at least one other mental health condition, with 60% and 45% presenting with two or three or more comorbid disorders, respectively [15]. These comorbidities can include mood disorders (e.g., Major Depressive Disorder—MDD, Bipolar Disorder—BD), anxiety disorders (e.g., Generalized Anxiety Disorder—GAD, Social Anxiety Disorder—SAD, Panic Disorder), substance use disorders, and personality disorders [17,18].

The presence of comorbid psychiatric conditions significantly impacts the course and severity of ADHD. This can manifest as increased functional impairment, encompassing difficulties in various domains of life, including work, relationships, and daily activities. Additionally, comorbidity increases the likelihood of experiencing a resurgence of ADHD symptoms, leading to a higher risk of relapse. Furthermore, the effectiveness of ADHD-specific interventions, such as medication and therapy, may be reduced in the presence of comorbid conditions, resulting in a poorer treatment response [19,20,21].

Given the significant impact of psychiatric comorbidities on adults with ADHD, it is crucial to understand how pharmacological treatments for ADHD affect these co-occurring conditions.

The treatment of adult ADHD often necessitates pharmacological interventions, primarily involving central nervous system stimulants or noradrenergic acting agents. This approach is well-supported by scientific literature and established guidelines [22]. Among the available options, Methylphenidate (MPH), a stimulant frequently employed as a first-line treatment since childhood, and Atomoxetine (ATX), a selective norepinephrine reuptake inhibitor, are most widely prescribed. Other medications, including Bupropion, a norepinephrine-dopamine reuptake inhibitor, Lisdexamfetamine (LDX), a prodrug stimulant that is metabolized into dextroamphetamine, and tricyclic antidepressants such as Imipramine and Desipramine, also find their place in treatment regimens [23,24,25].

This review aims to critically evaluate the existing evidence on the effectiveness of these pharmacological treatments for adult ADHD in addressing psychiatric comorbidity. The paper will focus specifically on the impact of the aforementioned commonly prescribed medications on the following comorbid conditions: mood disorders, anxiety disorders, personality disorders, substance use disorders, and disruptive disorders. Obsessive–Compulsive Disorder (OCD) was also considered a comorbid condition of interest a priori; however, as detailed in the Results and Discussion sections, no eligible studies reporting OCD-specific outcomes in adults with ADHD were identified. By synthesizing the existing research, this review seeks to provide clinicians with a comprehensive understanding of the potential benefits and limitations of pharmacological interventions for adult ADHD in the context of psychiatric comorbidity. This knowledge is essential for informing treatment decisions and developing personalized care plans that address the complex needs of this patient population.

## 2. Materials and Methods

A systematic review of published literature was conducted in accordance with the PICOS framework:

- P: The study population encompassed both female and male patients aged 18 years or older who met the established clinical criteria for ADHD and at least one psychiatric comorbidity.
- I: The intervention under investigation was psychopharmacological treatment for adults diagnosed with ADHD.
- C: The study considered comparisons between patients with ADHD and comorbidity before and after psychopharmacological treatment; matched groups treated with alternative forms of treatment (when available), control groups (when available).
- O: The primary outcome of interest was the change in the severity of general psychiatric symptoms.
- S: The review included a range of study designs, including randomized controlled trials, cohort studies, case–control studies, follow-up studies, pilot studies, quasi-experimental studies, case series, and case reports.

This systematic review was conducted in accordance with the Preferred Reporting Items for Systematic Reviews and Meta-Analyses guidelines [26]. This review was not registered with a research protocol.

Two authors independently searched PubMed for eligible articles. The search was limited to articles published in English. No lower date limit was imposed; PubMed was searched from database inception up to July 2025, and the earliest eligible article identified dated back to April 1994. The following search terms were used: (ADHD[tiab] OR “attention deficit hyperactivity disorder”[tiab] OR “attention-deficit hyperactivity disorder”[tiab] OR “attention-deficit/hyperactivity disorder”[tiab] OR “attention deficit/hyperactivity disorder”[tiab]) AND (comorb*[tiab] OR bipolar[tiab] OR personalit*[tiab] OR substanc*[tiab] OR anxiet*[tiab] OR panic[tiab]) AND (methylphenidat*[tiab] OR atomoxetin*[tiab] OR bupropio*[tiab] OR desipramin*[tiab] OR imipramin*[tiab] OR lisdexa*[tiab]).

To meet the inclusion criteria and encompass all original articles with a longitudinal design, whether prospective or retrospective, observational, experimental or quasi-experimental, controlled or non-controlled, the following filters were applied: Species: Humans; Language: English; Age: Adult (19+ years).

We did not extend the search to other bibliographic databases or gray literature sources. Therefore, this single-database search strategy should be regarded as a major methodological limitation and may have led to the omission of relevant studies, as discussed in Section 4.5.

Data extraction was carried out manually by two reviewers (B.T. and I.M.) working independently in a blinded manner, using a structured spreadsheet specifically developed for this review (template available as Supplementary Table S1). The spreadsheet contained predefined fields for study design, sample characteristics, interventions, outcomes, and main findings, and served as an internal data-extraction form. The reviewers collected information directly from the published articles and entered it into this spreadsheet. No dedicated electronic systematic-review management software was used. No study authors were contacted to obtain additional information or to clarify missing or unclear data. Any discrepancies in the extracted information were discussed and resolved by consensus, with the involvement of a third author (M.G.C.) when necessary.

During full-text screening, the reference lists of all included articles and of key narrative or systematic reviews on adult ADHD and psychiatric comorbidity were inspected to identify additional eligible studies, but this process did not yield further records meeting the inclusion criteria.

For each included study, data were extracted on both clinical outcomes and study-level characteristics. The primary outcome was the therapeutic response to ADHD-specific pharmacological treatments in adults with at least one psychiatric comorbidity, as reported in the original articles through validated clinical scales or investigator-rated assessments.

In addition to this primary endpoint, a comprehensive set of study characteristics was collected, as detailed in Table 1, Table 2, Table 3 and Table 4, which summarize the main characteristics of the included studies grouped by comorbidity domain (mood and anxiety disorders, personality disorders, substance use disorders, disruptive disorders). These variables included: first author and year of publication, study design, sample size and diagnostic features, type and dosage of the pharmacological intervention, presence and nature of comparator groups, psychometric instruments employed, and the main clinical outcomes reported. When available, information on follow-up duration, study setting, and measures of adherence or treatment compliance were also extracted.

No secondary outcomes were predefined beyond those captured within the included studies. All outcomes relevant to the research question were extracted without restrictions regarding specific measurement tools or time points. In cases where specific data elements were missing or not clearly reported, no assumptions were made.

The methodological quality of the studies included was assessed using the Joanna Briggs Institute (JBI) Critical Appraisal Tools, selecting the instrument appropriate for each study design. Two reviewers (B.T. and I.M.) independently evaluated the risk of bias for all included studies, working in a blinded manner and without knowledge of each other’s ratings. Any discrepancies were resolved through discussion with a third reviewer (M.G.C.).

The JBI appraisal considered key methodological domains, including clarity of participant selection, diagnostic ascertainment, appropriateness of the intervention and comparator, validity and reliability of outcome measures, completeness of follow-up, and transparency of reporting. Given the heterogeneity of study designs, variability in the overall risk of bias was expected. A detailed summary of the risk of bias assessments for each study is available in Supplementary Table S2.

Given the expected clinical and methodological heterogeneity across eligible studies (in terms of design, populations, psychometric instruments, and outcome definitions), we initially planned a primarily narrative synthesis of the evidence. A quantitative meta-analysis using random-effects models would have been undertaken if a sufficient number of studies with comparable designs, populations, and outcome measures had been identified for a given medication–comorbidity combination. However, as described in Section 3, the degree of between-study heterogeneity ultimately precluded statistical pooling, and no standardized effect measures (such as mean differences, standardized mean differences, risk ratios, or odds ratios) were calculated. All outcomes were therefore reported exactly as described by the original authors, including changes in validated clinical scales, categorical clinical judgments, or investigator-rated assessments.

The synthesis was developed by examining recurring patterns of treatment effects within and across the thematic groups, considering differences in study methodology, clinical characteristics, and assessment tools. No transformations, statistical pooling, or sensitivity analyses were undertaken, as these procedures were not applicable in the context of a narrative review.

## 3. Results

A total of 1257 records were initially identified through the PubMed search. Of these, 948 records were automatically excluded prior to screening through the built-in PubMed filters (Species: Humans; Language: English; Age: Adult 19+ years), which functioned as automation tools. After this automated step, 309 records remained.

**Figure 1:**
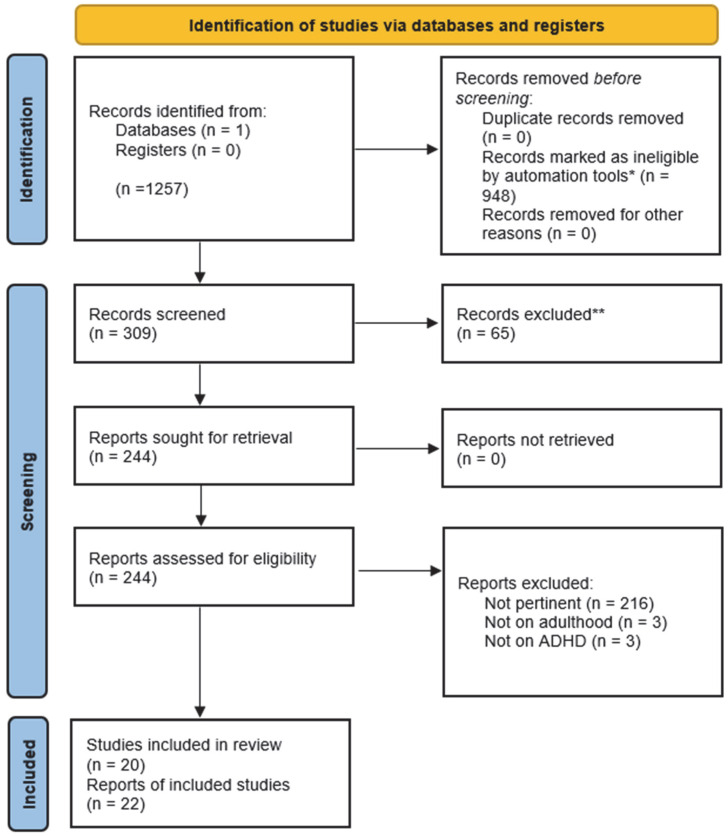
Flowchart of the study selection process. * All 948 records excluded prior to screening were removed through automated tools, specifically the built-in PubMed filters applied during the search. ** A total of 65 studies were excluded following human screening.

After removing reviews, meta-analyses, and non-original studies, 244 records proceeded to full screening. Of these, 216 records were excluded because they were not pertinent to the selected topic, 3 were excluded because the participants were younger than 18 years, and 3 were excluded because ADHD was not diagnosed. Ultimately, 20 studies met the inclusion criteria and were included in the qualitative synthesis, corresponding to a total of 22 reports of included studies (Figure 1).

The search yielded eligible studies investigating four pharmacological agents used in adult ADHD: Bupropion (*n* = 2), ATX (*n* = 4), MPH (*n* = 12), and LDX (*n* = 2). Findings are presented as a narrative synthesis. Due to the lack of homogeneity among the resulting studies in terms of design, populations, interventions, and outcome measures, a quantitative meta-analysis could not be performed.

Although tricyclic antidepressants (imipramine and desipramine) were included in the search strategy as potentially relevant pharmacological options [25], no longitudinal studies evaluating these agents in adults with ADHD and psychiatric comorbidity met the prespecified inclusion criteria. Likewise, Obsessive–Compulsive Disorder (OCD) was considered a comorbid condition of interest a priori, but none of the included studies reported OCD-specific outcomes or were designed to examine OCD as a primary or secondary endpoint. Consequently, these topics could not be addressed in the subsequent synthesis.

To facilitate interpretation, included studies were grouped into broad thematic categories based on their primary focus: affective and anxiety disorders, personality disorders, and substance use disorders. A single study addressing disruptive disorders in adults was reported separately due to its specificity. The main characteristics of the included studies are presented in four separate tables organized according to these comorbidity domains: Table 1 summarizes studies on mood and anxiety disorders, Table 2 those on personality disorders, Table 3 those on substance use disorders, and Table 4 the study on disruptive disorders. The same comorbidity-based structure is adopted in the narrative synthesis in Section 4.1, Section 4.2, Section 4.3 and Section 4.4.

Across the 20 included studies, 13 reported at least some improvement in the comorbid condition of interest associated with ADHD pharmacotherapy, 5 reported no clinically relevant change, and 2 yielded mixed findings, with benefits restricted to specific subgroups or anxiety dimensions.

When grouped by comorbidity domain, four out of nine studies focusing on mood and anxiety disorders described improvements in depressive and/or anxiety outcomes (with bupropion, ATX, or LDX), four randomized trials of MPH or ATX did not detect significant changes in mood or anxiety compared with placebo, and one crossover trial of MPH reported bidirectional effects on state anxiety depending on baseline anxiety levels. In the personality-disorder group, all three studies found improvements in borderline or broader personality-disorder symptoms or decision-making indices during MPH treatment. Among the seven studies targeting substance use disorders, five trials reported a reduction in substance use or craving for cocaine, amphetamine, or alcohol during treatment with bupropion, MPH, ATX, or LDX, whereas one trial in amphetamine dependence and one smoking-cessation trial did not show overall benefits, the latter suggesting possible benefit in patients with more severe ADHD and potential worsening in those with milder symptoms. The single randomized trial addressing disruptive disorders showed that MPH was superior to placebo in reducing oppositional defiant disorder symptoms in adults with comorbid ADHD and current ODD.

When outcomes are considered by medication, both studies investigating bupropion and both studies on LDX reported beneficial effects on at least one comorbidity-related outcome. Among the four ATX studies, three described improvements in social anxiety or alcohol craving, whereas one was neutral with respect to mood and anxiety symptoms. MPH was the most frequently investigated drug (14 reports): approximately half of these trials and pilot studies reported improvements in personality-disorder dimensions, disruptive behavior, or substance-use-related outcomes, four found no effect on mood, anxiety, or smoking cessation, and two yielded mixed or subgroup-dependent effects on anxiety or nicotine use.

According to the JBI Critical Appraisal Tools (Supplementary Table S2), the methodological quality of the included studies was heterogeneous. Overall, six studies were judged to have a low risk of bias, five a moderate risk, three a low–moderate risk, and six a high risk of bias, mostly due to non-randomized, open-label, or retrospective designs and incomplete control of confounding. High-risk studies were more common among older open-label or retrospective investigations of mood/anxiety and substance use disorders, whereas more recent randomized controlled trials, particularly those evaluating MPH and ATX, generally fell into the low or moderate risk categories.

Taking into account both the direction of effects and the risk-of-bias profile, the overall strength of the evidence should be considered limited. For descriptive purposes, we qualitatively classified the strength of evidence across comorbidity domains as preliminary (single small or high-risk-of-bias study), emerging (at least two studies with ≥1 randomized trial but important limitations), or moderate (multiple randomized trials with low or moderate risk of bias and broadly consistent results). Under this scheme, the evidence supporting pharmacological treatment of adults with ADHD and comorbid mood and anxiety disorders or substance use disorders is best described as emerging, given the presence of several randomized trials but heterogeneous and sometimes null findings. For personality disorders and for oppositional defiant disorder, the evidence remains preliminary, as conclusions rely on a small number of studies with short follow-up and, in some cases, higher risk of bias. No comorbidity–medication combination in this review is supported by a body of data that would justify a high level of confidence.

## 4. Discussion

Across comorbidity domains, a consistent pattern emerged: emotional dysregulation (ED) appears as a transdiagnostic construct that may partly account for the high co-occurrence of mood, anxiety, personality, substance use, and disruptive disorders in adults with ADHD. In this work, we therefore conceptualize ED as a potential psychopathological bridge between ADHD and its main psychiatric comorbidities, rather than as a simple secondary feature.

### 4.1. Comorbidity of ADHD and Affective Disorders, and Anxiety and Panic Spectrum Disorders

This section comprises nine studies that investigate the effects of ADHD medications on mood and anxiety disorders. The mood disorders primarily include MDD, Cyclothymia, and BD, while the anxiety disorders primarily include GAD and SAD.

Wilens et al. (2003) reported that sustained-release bupropion improved mood symptoms and, to a lesser extent, anxiety symptoms in adults with ADHD and various affective and anxiety comorbidities, although one patient developed hypomanic activation leading to treatment discontinuation [27]. These findings align with existing literature, which reports a potential for mild manic switching in bipolar patients treated with bupropion [49,50,51,52].

Similarly, McIntyre conducted a prospective study involving bipolar patients with ADHD and found that LDX use could improve depressive symptoms without inducing mood polarity switches over a short observation period [34].

In contrast to the findings with bupropion and LDX, a retrospective study by Reimherr et al. (2005) and a randomized controlled trial by Rösler et al. (2010) showed that, although ATX and MPH improved ADHD and ED symptoms, they did not produce significant changes in affective and anxiety symptoms compared to placebo [28,31]. These results support alternative nosological models that consider ED a core symptom of ADHD [53,54,55], potentially distinguishing between two subtypes: an “inattentive presentation” and an “emotional dysregulation presentation” [56].

Emotional dysregulation is estimated to be present in up to 70% of adults with ADHD [57]. Characterized by impaired executive or cognitive management of emotions, particularly those related to self-control of frustration, impatience, or anger, ED manifests as difficulty modulating behavior during specific emotional states. This difficulty appears to stem from biological underpinnings, such as hyperactivation of the amygdala or decreased inhibitory control in the prefrontal and orbitofrontal cortex.

Two distinct components of ED have been identified. Emotional Impulsivity (EI) is a bottom-up mechanism involving hyperactivation of mesolimbic circuits, including the amygdala, ventral striatum, and orbitofrontal cortex, and is defined as an increased likelihood of experiencing heightened reactions to primary emotions. Deficient Emotional Self-Regulation (DESR) is a top-down mechanism involving the ventrolateral prefrontal cortex (vlPFC), medial prefrontal cortex (mPFC), and anterior cingulate cortex, and is characterized by a deficit in regulating emotional intensity and generating secondary emotions to counteract initial emotional responses [58].

ED appears to be a predisposing and risk factor for developing other psychiatric disorders, including Cluster B and C personality disorders, mood disorders, anxiety disorders, behavioral disorders, and substance use disorders [6,16,59,60].

From a clinical perspective, our findings support the routine assessment of ED in adult ADHD, using specific questions or rating scales, and suggest that pharmacological treatment should be planned and monitored taking individual ED profiles into account. In patients with marked ED and comorbid mood, personality, or substance use disorders, ADHD medication may be more effective when combined with psychotherapeutic interventions explicitly targeting emotion regulation (e.g., CBT- or DBT-informed approaches).

Consistent with the studies mentioned above, two 6-week randomized controlled trials from the same research group, involving a large sample of adults with ADHD and comorbid affective and/or anxiety disorders, did not find a significant effect of MPH on mood or anxiety compared with placebo [29,32]. In contrast, Adler et al. (2009) observed a significant improvement in social anxiety scores after ATX treatment compared to placebo, although this effect was not confirmed when stratifying the sample by the presence of GAD [30]. Gabriel and Violato (2011) further explored the relationship between ATX and anxiety in patients with ADHD and GAD, suggesting that ATX may improve predominantly cognitive anxiety symptoms and that anxiety may partly arise from repeated failures and frustrations associated with ADHD-related challenges in daily life [33].

Segev et al. (2016) provided more nuanced findings. In a crossover RCT, they showed that, after stratifying the sample by baseline state and trait anxiety, MPH administration decreased both types of anxiety in individuals with high initial state anxiety, whereas state (but not trait) anxiety worsened in those with low initial state anxiety [35].

Overall, the research on the effects of ADHD medication on mood and anxiety presents a complex picture with contrasting results. While these medications aim to address dopamine and norepinephrine dysfunction, a key hypothesis underlying ADHD, their impact extends beyond this mechanism [61,62,63]. Bupropion shows promise in improving mood disorders in individuals with ADHD and comorbid BD and anxiety disorders, but carries a risk of mood destabilization in susceptible individuals [64].

Evidence for LDX is still limited but suggests potential antidepressant effects without clear mood switches. ATX and MPH show heterogeneous effects on mood and anxiety, with possible benefits for specific anxiety phenotypes and a risk of exacerbating anxiety in others [65]. Further research is needed to clarify which patient subgroups are most likely to benefit or worsen, and to elucidate the mechanisms by which ADHD medications influence mood and anxiety.

### 4.2. Comorbidity of ADHD and Personality Disorders

This section delves into the complex relationship between ADHD and personality disorders, specifically focusing on Borderline Personality Disorder (BPD), as evidenced by three detailed studies. While other research acknowledged the presence of personality disorders within their samples, the lack of in-depth analysis led to their exclusion from this section.

A key psychopathological element shared by both ADHD and personality disorders, particularly BPD, is ED. As discussed in the previous section, certain medications, by effectively managing ED, may offer a promising avenue for treating both conditions when comorbid [31]. This potential overlap is further supported by the dopaminergic dysfunction hypothesis, suggesting a common biological underpinning for both ADHD and BPD, which might explain the observed benefits of stimulant medication in both disorders [66].

A small N-of-1 study showed that MPH reduced core borderline symptoms in a patient with comorbid ADHD and BPD, including mood swings, suicidal ideation, reckless behaviors, self-harm urges, and anxiety [36]. In a larger sample, Gift et al. (2016) found that long-term treatment with MPH in adults with ADHD and personality disorders was associated with improvement in both ADHD and personality-disorder symptomatology, with particularly marked changes in borderline and narcissistic traits across several psychometric scales [37].

A crossover RCT in adults with ADHD and BPD further suggested that MPH can improve decision-making, a cognitive process often impaired in BPD [67,68]. Interestingly, the degree of improvement was inversely correlated with the severity of inattention symptoms, indicating that patients with less severe inattention may derive greater benefit in this specific domain [38].

In conclusion, while the use of MPH appears to offer some benefits for managing personality disorders in the context of ADHD, it is crucial to acknowledge that comorbidity between these disorders has been linked to a significant reduction in treatment response rates and improvement in inattentive symptoms [69,70,71]. This highlights the need for further research to develop personalized treatment approaches for individuals with ADHD and comorbid personality disorders.

### 4.3. Comorbidity of ADHD and Substance Use Disorder

This section summarizes seven articles exploring the complex relationship between ADHD and substance use disorders, specifically focusing on cocaine dependence, amphetamine dependence, alcohol abuse, and smoking.

#### 4.3.1. ADHD Medication and Cocaine Dependence

Pilot and open-label studies by Levin and colleagues and Somoza et al. suggest that pharmacological treatment of ADHD with bupropion or MPH may reduce ADHD symptoms and cocaine use or craving in adults with dual diagnosis of ADHD and cocaine dependence [39,40,41]. A more recent randomized, placebo-controlled trial of LDX in adults with ADHD and comorbid cocaine use disorder found a partial reduction in cocaine use and a significant decrease in craving over the treatment period, indicating a possible but not definitive benefit [47].

#### 4.3.2. ADHD Medication and Amphetamine Dependence

Two RCTs by Konstenius and colleagues evaluated sustained-release MPH in adults with ADHD and amphetamine dependence. In the first study, MPH did not significantly differ from placebo in terms of relapse rate or cumulative abstinence [42]. In the second, conducted in criminal offenders with substance dependence, MPH was associated with a longer time to first positive urine test and lower craving compared with placebo [44].

#### 4.3.3. ADHD Medication and Alcohol and Nicotine Use

In adults with ADHD and alcohol use disorder, a placebo-controlled trial of ATX reported a significant reduction in alcohol craving in the active-treatment group, with improvements directly correlating with changes in ADHD symptoms [43]. Finally, Luo et al. reanalyzed data from a randomized trial of MPH as an adjunct to smoking-cessation treatment in individuals with ADHD [46]. Their predictive modeling suggested that patients with more severe ADHD may achieve prolonged abstinence with MPH, whereas those with less severe ADHD could experience poorer outcomes, underscoring the importance of individual differences when considering stimulant treatment in smokers with ADHD [45].

The research on the effectiveness of ADHD medication in reducing substance use and cravings presents a mixed picture. While some studies suggest potential benefits, particularly for cocaine and alcohol use disorders, others show limited or even negative effects. The findings regarding amphetamine dependence and smoking cessation are inconclusive and highlight the need for further research to understand the complex interplay of individual factors, medication type, and specific substance use disorder. Larger, well-controlled studies with longer follow-up periods are crucial to determine the long-term efficacy and safety of ADHD medication in managing substance use disorders in individuals with ADHD.

### 4.4. Comorbidity of ADHD and Disruptive Disorders

Disruptive disorders in childhood and adolescence encompass a range of conditions characterized by persistent patterns of disruptive and challenging behaviors that deviate significantly from age-appropriate norms. These disorders, such as Oppositional Defiant Disorder (ODD) and Conduct Disorder (CD), often involve difficulties in emotional and behavioral regulation, leading to conflicts with authority figures and peers, and can significantly impair a child’s social, academic, and family functioning. It is important to note the significant symptom overlap between ADHD and disruptive disorders, particularly the shared difficulties in impulse control and emotional regulation. This overlap suggests a potential common neurobiological basis for these conditions, warranting further investigation [72].

While several studies mentioned above included individuals with disruptive disorders in their samples, only one specifically investigated the effects of MPH therapy on this comorbidity. This crossover randomized controlled trial by Reimherr et al. (2013) [48] involved three groups of adult patients: 36 adults with ADHD and current ODD, 23 adults with ADHD and a childhood history of ODD (assessed retrospectively), and 27 adults with ADHD who never met the criteria for ODD. The study found that MPH was superior to placebo in improving ODD symptoms specifically in the group with current comorbid ADHD and ODD [48]. These findings suggest that MPH may hold promise in addressing not only the core symptoms of ADHD but also the challenging behaviors associated with ODD in individuals with both conditions. However, it is crucial to acknowledge the limitations of a single study. Further research, including larger-scale RCTs with longer follow-up periods, is necessary to replicate these findings, confirm the efficacy and safety of MPH in treating comorbid ADHD and ODD across different age groups, and determine the long-term impact of such treatment on both conditions.

**Figure 2:**
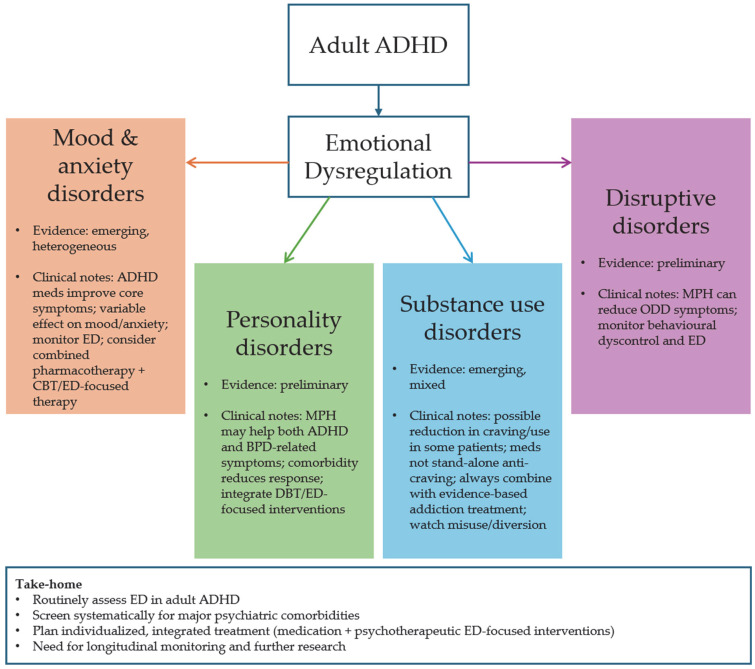
Clinical implications of pharmacological treatment for adults with ADHD and psychiatric comorbidities. Schematic representation of the main clinical implications derived from this systematic review for the pharmacological management of adults with ADHD and psychiatric comorbidities. Emotional dysregulation (ED) is conceptualized as a transdiagnostic bridge between ADHD and major comorbidity domains. For each domain, the figure qualitatively summarizes the current strength of evidence (preliminary vs. emerging) and key practical considerations for integrating pharmacological treatment with broader clinical management (e.g., routine assessment of ED, combination with psychotherapeutic interventions, and integration with addiction treatment services in the case of substance use disorders). ODD = oppositional defiant disorder; BPD = borderline personality disorder.

Figure 2 provides a schematic overview of these clinical implications, highlighting emotional dysregulation as a potential transdiagnostic bridge between ADHD and comorbid conditions and outlining practical considerations for integrating pharmacological treatments into broader, individualized care plans.

### 4.5. Limitations

This review has several methodological limitations that should be acknowledged. First, the literature search was conducted exclusively on PubMed. Although PubMed provides broad coverage of the biomedical literature, reliance on a single database represents a major limitation for a systematic review and increases the risk of missing relevant studies, including those indexed in other databases (e.g., Embase, PsycINFO, Web of Science) or in the gray literature. As a result, the body of evidence synthesized here may not fully capture all available data on pharmacological treatment of adult ADHD with psychiatric comorbidities, and our conclusions should be interpreted in light of this potential selection and publication bias. Second, the review was not registered in PROSPERO or any other registry, and no formal protocol was prepared, introducing potential risks of methodological drift. Third, data extraction was performed manually using an ad hoc spreadsheet, without the support of dedicated systematic-review software or a previously validated, standardized extraction form. This increases the possibility of human error and may limit reproducibility despite the use of independent reviewers; to enhance transparency, a template of the extraction spreadsheet has been provided as Supplementary Table S1. Although the methodological quality of the included studies was assessed using the appropriate Joanna Briggs Institute (JBI) Critical Appraisal Tools, no formal assessment of reporting bias or certainty of evidence was performed. Finally, the decision not to conduct a quantitative synthesis further limits reproducibility and interpretability of the findings. These factors should be considered when interpreting the conclusions of this review.

## 5. Conclusions

Several studies have delved into the intricate relationship between ADHD, psychiatric comorbidities, and the effects of stimulant or noradrenergic medications. While these medications consistently demonstrate efficacy in ameliorating core ADHD symptoms, such as inattention, hyperactivity, and impulsivity, and show promise in improving executive function, their impact on co-occurring psychiatric conditions presents a more nuanced picture.

The efficacy of these medications on mood and affective symptoms remains ambiguous. While some studies suggest potential mood-stabilizing effects, particularly in individuals with comorbid bipolar disorder, others report potential for mood destabilization or the exacerbation of anxiety. This variability likely stems from a complex interplay of factors, including individual patient characteristics, medication type and dosage, and the specific nature of the comorbid mood or anxiety disorder. For instance, individuals with pre-existing anxiety disorders, particularly those characterized by high physiological arousal, might be more susceptible to experiencing heightened anxiety with stimulant medications.

Emerging evidence suggests a potential positive impact on personality disorders, particularly those within Cluster B, which share common underlying neurobiological pathways with ADHD. Specifically, the ability of these medications to improve emotional regulation and impulsivity might contribute to their observed benefits in managing borderline personality disorder. However, it is crucial to acknowledge that not all personality disorders demonstrate the same degree of benefit, and further research is needed to determine the optimal treatment approaches for specific subtypes.

Regarding substance use disorders, the data indicate a potential reduction in craving across various substances, including alcohol, cocaine, and nicotine. This effect might be attributed to the medications’ influence on dopamine pathways in the brain, which are implicated in reward and craving mechanisms. However, maintaining this efficacy over the long term presents a significant challenge, and relapse rates remain high. Integrating these medications within a comprehensive treatment plan that addresses the multifaceted nature of addiction, including individual and environmental factors, is crucial for optimizing long-term outcomes. The heterogeneity of existing data, including variations in study designs, sample characteristics, medication regimens, and the specific psychiatric comorbidities investigated, necessitates cautious interpretation of the findings. Larger, well-controlled studies with longer follow-up periods are essential to definitively determine the long-term efficacy and safety of these medications in managing both core ADHD symptoms and the diverse range of co-occurring psychiatric conditions. Additionally, personalized treatment approaches that consider individual patient characteristics, comorbidity profiles, and potential risks and benefits are paramount to optimize outcomes and minimize adverse effects.

In navigating the intricate interplay between ADHD and co-occurring psychiatric conditions, a nuanced and individualized approach to treatment is paramount. While stimulant or noradrenergic medications offer valuable tools for managing core ADHD symptoms and show promise in addressing certain comorbidities, their impact is not uniform across all individuals or psychiatric profiles. Careful consideration of individual patient characteristics, comorbidity profiles, potential risks and benefits, and long-term treatment goals is essential to optimize outcomes and provide holistic care.

## Figures and Tables

**Table 1:** Mood and anxiety disorders (*n* = 9).

First Author and Year	Study Design	Subjects and Diagnoses	Pharmacotherapy	Comparisons	Outcomes
Wilens, T. E., 2003 [27]	Open-label, prospective, 6-week	36 ADHD and BD	Bupropion (up to 400 mg/day)	CGI, ARS, HAM-D, BDI, HAM-A, YMRS	Reduction in manic and depressive symptoms. Mild reduction in anxiety.
Reimherr, F. W., 2005 [28]	Retrospective	536 ADHD and affective and/or anxiety disorders	ATX (up to 120 mg/day)	CGI, CAARS, WRAADDS, HAM-D, HAM-A, SDS	↑ Emotional regulation in ATX vs. placebo. No significant changes in depression or anxiety between ATX and placebo.
Spencer, T., 2005 [29]	Randomized, double-blind, placebo-controlled, parallel-design, 6-week	146 ADHD and affective and/or anxiety disorders	MPH (*n* = 104) (mean 82 ± 22 mg/day) Placebo (*n* = 42)	CGI, AISRS, HAM-D, BDI; HAM-A	No significant changes in depression or anxiety between MPH and placebo.
Adler, L. A., 2009 [30]	Randomized, double-blind, placebo-controlled, parallel-design, multi-center, 16-week	442 ADHD and social anxiety and/or GAD	ATX (*n* = 224) (up to 100 mg/day) Placebo (*n* = 218)	LSAS, CGI, CAARS:Inv:SV, SAS, STAI	↑ ADHD and social anxiety symptoms in ATX vs. placebo. No significant changes in GAD between ATX and placebo.
Rösler, M., 2010 [31]	Randomized, double-blind, placebo-controlled, parallel-design, multi-center, 24-week	363 ADHD and affective, anxiety, and/or personality disorders	MPH (*n* = 241) (up to 60 mg/day) Placebo (*n* = 118)	WRAADDS, CAARS-S:L, SCL-90-R	↑ Emotional regulation in MPH vs. placebo. No significant changes in depression or anxiety between MPH and placebo.
Biederman, J., 2012 [32]	Randomized, double-blind, placebo-controlled, parallel-design, 6-week	227 ADHD and affective, anxiety disorders, and/or SUD	MPH (*n* = 112) (mean 78.4 ± 31.7 mg/day) Placebo (*n* = 115)	CGI, AISRS, HAM-D, HAM-A, GAF	No significant changes in depression, anxiety, or SUD between MPH and placebo.
Gabriel, A., 2011 [33]	Open-label, cross-sectional, 12-week	29 ADHD and GAD	ATX (up to 80 mg/day)	CGI, ASRS, HAM-A, SDS, CADDRA side effect scale	↑ Anxiety symptoms. Cognitive anxiety symptoms are predominant over the somatic anxiety symptoms.
McIntyre, R. S., 2013 [34]	Open-label, prospective, flexible-dose, 4-week	40 ADHD and BD	LDX (up to 70 mg/day)	CGI, YMRS, MADRS, C-SSRS, ADHD-RS, CAARS, Q-LES-Q, AAQoL	Less MADRS total score. No induction of hypo/manic or psychotic symptomatology.
Segev, A., 2016 [35]	Randomized, double-blind, placebo-controlled, random block order crossover, two sessions 2 weeks apart	36 ADHD and anxiety disorders	MPH (20 mg/session) Placebo	STAI, VAS	↑ state and trait-anxiety in “high initial state-anxiety” group with MPH. ↓ state-anxiety in “low initial state-anxiety” group with MPH. No changes with placebo.

Abbreviations: ↑: better; ↓: worse; AAQoL: Adult ADHD Quality of Life Scale; ADHD: Attention-Deficit and Hyperactivity Disorder; ADHD-RS: Attention Deficit Hyperactivity Disorder Rating Scale; AISRS: Adult ADHD Investigator System report Scale; ARS: ADHD Rating Scale; ASRS: Adult Self-Report Scale; ATX: Atomoxetine; BD: Bipolar Disorder; BDI: Beck Depression Inventory; BDI-II: Beck Depression Inventory, 2nd Edition; CAARS: Conners’ Adult ADHD Rating Scale; CAARS:Inv:SV: Conners’ Adult ADHD Rating Scale: Investigator-Rated: Screening Version; CAARS-S:L: Conners’ Adult ADHD Rating Scale Self Report Long Form; CADDRA: Canadian ADHD Resource Alliance; CGI: Clinical Global Impression; C-SSRS: Columbia-Suicide Severity Rating Scale; GAD: Generalized Anxiety Disorder; GAF: Global Assessment of Functioning; HAM-A: Hamilton Anxiety Scale; HAM-D: Hamilton Depression Scale; LDX: Lisdexamphetamine; LSAS: Liebowitz Social Anxiety Scale; MADRS: Montgomery-Åsberg Depression Rating Scale; MPH: Methylphenidate; Q-LES-Q: Quality of Life Enjoyment and Satisfaction Questionnaire; SAS: Social Adjustment Scale-Self Report; SCL-90-R: Symptom Checklist 90-Revised; SDS: Sheehan Disability Scale; STAI: State-Trait Anxiety Inventory; VAS: Visual Analog Scale; WRAADDS: Wender-Reimherr Adult Attention Deficit Disorder Scale; YMRS: Young Mania Rating Scale.

**Table 2:** Personality disorders (*n* = 3).

First Author and Year	Study Design	Subjects and Diagnoses	Pharmacotherapy	Comparisons	Outcomes
van Reekum, R., 1994 [36]	N of 1, randomized, double-blind, placebo-controlled, 18-week	1 ADHD and BPD	MPH (up to 40 mg/day) Placebo	Symptoms, attention, vigilance, and impulse inhibition assessments, urine test	Significant decrease of BPD symptomatology in MPH vs. placebo.
Gift, T. E., 2016 [37]	Two randomized, double-blind, placebo-controlled, flexible-dose, crossover, two 4-week and subsequently 6-month open-label phase	115 ADHD and personality disorders	MPH Placebo	CGI, WRAADS, WISPI-IV	↑ personality disorder’s symptoms in end-point vs. baseline. Narcissistic and Borderline D. showed improvement in more items of the WISPI-IV than the other disorders. Measures were not correlated with changes in ADHD.
Gvirts, H. Z., 2018 [38]	Randomized, double-blind, placebo-controlled, crossover, two session 1–2 weeks apart	20 ADHD and BPD	MPH (20–30 mg/session) Placebo	TOVA, forward and backward digit-span tasks, IGT	Lower scores of inattention symptoms were associated with greater improvement in decision-making in MPH vs. placebo

Abbreviations: ↑: better; ADHD: Attention-Deficit and Hyperactivity Disorder; BPD: Borderline Personality Disorder; CGI: Clinical Global Impression; IGT: Iowa Gambling Task; MPH: Methylphenidate; TOVA: Test of Variables of Attention; WISPI-IV: Wisconsin Personality Disorders Inventory IV; WRAADDS: Wender-Reimherr Adult Attention Deficit Disorder Scale.

**Table 3:** Substance use disorders (*n* = 7).

First Author and Year	Study Design	Subjects and Diagnoses	Pharmacotherapy	Comparisons	Outcomes
Levin, F. R., 2002 [39] (including Levin, F. R., 1998 [40])	Cross-sectional, 12-week	10 + 10 ADHD and CUD	Bupropion (*n* = 10) (up to 400 mg/day) MPH (*n* = 10) (up to 80 mg/day)	ARS, TADDS, ASI, urine test	↑ ADHD symptoms, cocaine-free weeks, and less cocaine craving in Bupropion and MPH, the efficacy is comparable.
Somoza, E. C., 2004 [41]	Open-label, multi-center, 10-week	41 ADHD and CUD	MPH (up to 60 mg/day) Compliant (*n* = 19) Non-compliant (*n* = 22)	CGI, ASI, SUQ, BSCS, CCQ-GEN, RAB, urine test, CalCAP	Low level of cocaine use in compliant vs. non-compliant.
Konstenius, M., 2010 [42]	Randomized, double-blind, placebo-controlled, 12-week	24 ADHD and amphetamine dependence	MPH (*n* = 12) (up to 72 mg/day) Placebo (*n* = 12)	CAARS:SV, CAARS-O, ASI, TLFB, Tiffany Craving scale, BDI-II, BAI, Stroop Test, urine test	No significant changes in relapse rate, time to relapse or cumulative abstinence rate between MPH and placebo.
Wilens, T. E., 2011 [43]	Randomized, double-blind, placebo-controlled, multi-center, 12-week	147 ADHD and alcohol abuse	ATX (*n* = 72) (up to 100 mg/day) Placebo (*n* = 75)	AISRS, ASRS, Timeline Followback method, OCDS	Reduction in alcohol craving correlated with improvements in ADHD symptoms
Konstenius, M., 2014 [44]	Randomized, double-blind, placebo-controlled, parallel-design, 24-week	54 ADHD and amphetamine dependence	MPH (*n* = 27) (up to 180 mg/day) Placebo (*n* = 27)	CGI, CAARS:SV, OQ45, urine test	Shorter time to first positive urine in placebo vs. MPH. Significantly decrease of craving in MPH vs. placebo.
Luo, S. X., 2015 [45] (including Winhusen, T. M., 2010 [46])	Randomized, double-blind, placebo-controlled, parallel-design, multi-center, 11-week	255 ADHD and smokers	MPH (*n* = 127) (up to 72 mg/day) Placebo (*n* = 128)	Expired carbon monoxide, MNWS, FTND, BDI, ADHD-RS	No significant improvement in smoking cessation in MPH vs. placebo. MPH may promote prolonged abstinence for more severe ADHD (ADHD-RS > 35), conversely it may be counterproductive.
Mooney, M. E., 2015 [47]	Randomized, double-blind, placebo-controlled, parallel-design, 14-week	43 ADHD and CUD	LDX (*n* = 22) (up to 70 mg/day) Placebo (*n* = 21)	BDI-II, ASI, urine test	Partial evidence of reduction in cocaine use in LDX vs. placebo. Less cocaine craving in LDX vs. placebo.

Abbreviations: ↑: better; ADHD: Attention-Deficit and Hyperactivity Disorder; ADHD-RS: Attention Deficit Hyperactivity Disorder Rating Scale; AISRS: Adult ADHD Investigator System report Scale; ARS: ADHD Rating Scale; ASI: Addiction Severity Index; ASRS: Adult Self-Report Scale; ATX: Atomoxetine; BAI: Beck Anxiety Inventory; BDI: Beck Depression Inventory; BDI-II: Beck Depression Inventory, 2nd Edition; BSCS: Brief Substance Craving Scale; CAARS:SV: Conners’ Adult ADHD Rating Scale: Screening Version; CAARS-O: Conners’ Adult ADHD Rating Scale-Observer; CCQ-GEN: Cocaine Craving Questionnaire-General; CGI: Clinical Global Impression; CUD: Cocaine Use Disorder; CalCAP: California Computerized Assessment Package; FTND: Fagerstrom Test for Nicotine Dependence; LDX: Lisdexamphetamine; MNWS: Minnesota Nicotine Withdrawal Symptoms; MPH: Methylphenidate; OCDS: Obsessive Compulsive Drinking Scale; RAB: Risk Assessment Battery; SUD: Substance Use Disorder; SUQ: Substance Use Questionnaire; TADDS: Targeted Attention Deficit Disorder Symptom; TLFB: Time-Line Follow-Back self-report interview.

**Table 4:** Disruptive disorders (*n* = 1).

First Author and Year	Study Design	Subjects and Diagnoses	Pharmacotherapy	Comparisons	Outcomes
Reimherr, F. W., 2013 [48]	Randomized, double-blind, placebo-controlled, crossover, two 4-week	36 ADHD and adult ODD 23 ADHD and child ODD 27 ADHD non-ODD	MPH (up to 30 mg/day) Placebo	CGI, WRAADS, CAARS, DBRS	↑ ODD symptomatology in ADHD and ODD group, highly correlated with changes in ADHD symptoms

Abbreviations: ↑: better; ADHD: Attention-Deficit and Hyperactivity Disorder; CAARS: Conners’ Adult ADHD Rating Scale; CGI: Clinical Global Impression; DBRS: Disruptive Behavior Rating Scale; MPH: Methylphenidate; ODD: Oppositional Defiant Disorder; WRAADDS: Wender-Reimherr Adult Attention Deficit Disorder Scale.

## Data Availability

All data extracted for this review are included within the manuscript and its Supplementary Materials. In addition, a template of the spreadsheet used for data extraction is provided as Supplementary Table S1 to facilitate transparency and reproducibility. No additional datasets or analysis code were generated.

## Supplementary Materials

The following supporting information can be downloaded at: https://www.mdpi.com/article/10.3390/jcm14248848/s1, PRISMA 2020 Checklist [26]. Table S1. Data extraction template for included studies. Table S2. Risk of bias assessment of the included studies using the Joanna Briggs Institute (JBI) Critical Appraisal Tools.

## Abbreviations

The following abbreviations are used in this manuscript:

↑	better
↓	worse
AAQoL	Adult ADHD Quality of Life Scale
ADHD	Attention-Deficit/Hyperactivity Disorder
ADHD-RS	Attention-Deficit/Hyperactivity Disorder Rating Scale
AEP	Liverpool Adverse Events Profile
AES	Apathy Evaluation Scale
AISRS	Adult ADHD Investigator Symptom Report Scale
ARS	ADHD Rating Scale
ARS-IV	ADHD Rating Scale-IV
ASI	Addiction Severity Index
ASD	Autism Spectrum Disorder
ASRS	Adult Self-Report Scale
ATX	Atomoxetine
BAI	Beck Anxiety Inventory
BD	Bipolar Disorder
BDI	Beck Depression Inventory
BDI-II	Beck Depression Inventory, 2nd Edition
BPD	Borderline Personality Disorder
BSCS	Brief Substance Craving Scale
CAARS	Conners’ Adult ADHD Rating Scale
CAARS:Inv:SV	Conners’ Adult ADHD Rating Scale: Investigator-Rated: Screening Version
CAARS:SV	Conners’ Adult ADHD Rating Scale: Screening Version
CAARS-O	Conners’ Adult ADHD Rating Scale-Observer
CAARS-S:L	Conners’ Adult ADHD Rating Scale Self-Report Long Form
CADDRA	Canadian ADHD Resource Alliance
CalCAP	California Computerized Assessment Package
CCQ-GEN	Cocaine Craving Questionnaire–General
CD	Conduct Disorder
CGI	Clinical Global Impression
CPT3	Conners’ Continuous Performance Test, 3rd Edition
C-SSRS	Columbia-Suicide Severity Rating Scale
CUD	Cocaine Use Disorder
DBRS	Disruptive Behavior Rating Scale
DESR	Deficient Emotional Self-Regulation
ED	Emotional Dysregulation
EI	Emotional Impulsivity
FTND	Fagerström Test for Nicotine Dependence
GAD	Generalized Anxiety Disorder
GAF	Global Assessment of Functioning
GWB	General Well-Being Schedule
HAM-A	Hamilton Anxiety Scale
HAM-D	Hamilton Depression Scale
IGT	Iowa Gambling Task
LDX	Lisdexamfetamine
LSAS	Liebowitz Social Anxiety Scale
MADRS	Montgomery–Åsberg Depression Rating Scale
MCG	Medical College of Georgia Paragraph Memory Test
MDD	Major Depressive Disorder
MNWS	Minnesota Nicotine Withdrawal Symptoms
MPH	Methylphenidate
OCD	Obsessive–Compulsive Disorder
OCDS	Obsessive Compulsive Drinking Scale
ODD	Oppositional Defiant Disorder
OQ45	Outcome Questionnaire-45
PICOS	Population, Intervention, Comparison, Outcome, Study Design
PRISMA	Preferred Reporting Items for Systematic Reviews and Meta-Analyses
Q-LES-Q	Quality of Life Enjoyment and Satisfaction Questionnaire
QOLIE-89	Quality of Life in Epilepsy-89
RAB	Risk Assessment Battery
SAD	Social Anxiety Disorder
SAS	Social Adjustment Scale–Self-Report
SCL-90-R	Symptom Checklist-90–Revised
SCWT	Stroop Color–Word Test
SDMT	Symbol Digit Modalities Test
SDS	Sheehan Disability Scale
SSC	Stimulant Side Effect Checklist
STAI	State-Trait Anxiety Inventory
SUD	Substance Use Disorder
SUQ	Substance Use Questionnaire
TADDS	Targeted Attention Deficit Disorder Symptom Scale
TLFB	Time-Line Follow-Back self-report interview
TOVA	Test of Variables of Attention
VAS	Visual Analog Scale
WISPI-IV	Wisconsin Personality Disorders Inventory-IV
WRAADDS	Wender–Reimherr Adult Attention Deficit Disorder Scale
YMRS	Young Mania Rating Scale
