# Supplementary material for: Effectiveness of Pharmacological Treatments for Adult ADHD on Psychiatric Comorbidity: A Systematic Review
Source: J Clin Med. 2025 Dec 14;14(24):8848. doi: 10.3390/jcm14248848 (PMC12734097; doi:10.3390/jcm14248848)
Supplement: Supplementary file 1 [file jcm-14-08848-s001.zip › Supplemetary Table 1.pdf]

Table S1. Data extraction template for included studies.

First author	Year of publication	Country / setting	Study design	Sample size	Diagnostic criteria and instruments for ADHD	Psychiatric comorbidities	Intervention	Comparator / control condition	Primary outcomes	Secondary outcomes / adverse events	Follow-up duration	Notes / comments
