# Supplementary material for: Effectiveness of Pharmacological Treatments for Adult ADHD on Psychiatric Comorbidity: A Systematic Review
Source: J Clin Med. 2025 Dec 14;14(24):8848. doi: 10.3390/jcm14248848 (PMC12734097; doi:10.3390/jcm14248848)
Supplement: Supplementary file 1 [file jcm-14-08848-s001.zip › Supplemetary Table 2.pdf]

Table S2. Risk of bias assessment of the included studies using the Joanna Briggs Institute (JBI) Critical Appraisal Tools

Study (Year)	Design	Clear inclusion criteria	Randomization / group comparability	Blinding (participants / assessors)	Valid & reliable outcome measures	Attrition adequately handled	Confounding addressed	Statistics appropriate	Overall Risk of Bias
van Reekum (1994)	N-of-1 randomized, double-blind	Y	Y	Y	Y	Y	Y	Y	Low–Moderate
Levin (2002/1998)	Comparative, non-randomized	Y	N	N	Y	Y	N	Y	High
Wilens (2003)	Open-label prospective	Y	N	N	Y	Y	N	Y	High
Somoza (2004)	Open-label, multicenter	Y	N	N	Y	Y	N	Y	High
Reimherr (2005)	Retrospective cohort	Y	N	U	Y	N	N	Y	High
Spencer (2005)	RCT, double-blind, placebo-controlled	Y	Y	Y	Y	Y	Y	Y	Low
Adler (2009)	RCT, double-blind, multicenter	Y	Y	Y	Y	Y	Y	Y	Low
Konstenius (2010)	RCT, double-blind	Y	Y	Y	Y	Y	Y	Y	Moderate

Rösler (2010)	RCT, double-blind, multicenter	Y	Y	Y	Y	Y	Y	Y	Low
Biederman (2011)	RCT, double-blind	Y	Y	Y	Y	Y	Y	Y	Low
Gabriel (2011)	Open-label	Y	N	N	Y	Y	N	Y	High
Wilens (2011)	RCT, double-blind	Y	Y	Y	Y	Y	Y	Y	Low
McIntyre (2013)	Open-label	Y	N	N	Y	Y	N	Y	High
Reimherr (2013)	RCT, double-blind, crossover	Y	Y	Y	Y	Y	Y	Y	Low
Konstenius (2014)	RCT, double-blind	Y	Y	Y	Y	Y	Y	Y	Moderate
Luo / Winhusen (2015/2010)	RCT, double-blind, multicenter	Y	Y	Y	Y	Y	Y	Y	Low–Moderate
Mooney (2015)	RCT, double-blind	Y	Y	Y	Y	Y	Y	Y	Low–Moderate
Gift (2016)	Two RCTs + open-label extension	Y	Y	Y/N	Y	Y	Y	Y	Moderate

Segev (2016)	RCT, double-blind, crossover	Y	Y	Y	Y	Y	Y	Y	Moderate
Gvirts (2018)	RCT, double-blind, crossover	Y	Y	Y	Y	Y	Y	Y	Moderate

Y = Yes, criterion fulfilled; N = No, criterion not fulfilled; U = Unclear / Not reported, insufficient information provided in the original publication; RCT = Randomized Controlled Trial. Overall risk-of-bias judgments were based on the pattern of domain ratings for each study: Low: most domains satisfy criteria, no critical weaknesses; Moderate: some limitations present but unlikely to substantially affect validity; High: significant methodological limitations likely to introduce bias.
